# Current and future environmental suitability for bats hosting potential zoonotic pathogens in rural Kenya

**DOI:** 10.1002/ece3.11572

**Published:** 2024-06-14

**Authors:** Ruut J. Uusitalo, Reilly T. Jackson, Tamika J. Lunn, Essi M. Korhonen, Joseph G. Ogola, Paul W. Webala, Tarja A. Sironen, Kristian M. Forbes

**Affiliations:** ^1^ Department of Virology, Faculty of Medicine University of Helsinki and Helsinki University Hospital Helsinki Finland; ^2^ Department of Geosciences and Geography, Faculty of Science University of Helsinki Helsinki Finland; ^3^ Department of Veterinary Biosciences, Faculty of Veterinary Medicine University of Helsinki Helsinki Finland; ^4^ Department of Biological Sciences, Fullbright College of Arts and Sciences University of Arkansas Fayetteville Arkansas USA; ^5^ Arizona Game and Fish Department Wildlife Research Branch Phoenix Arizona USA; ^6^ Odum School of Ecology University of Georgia Athens USA; ^7^ Center for the Ecology of Infectious Diseases University of Georgia Athens USA; ^8^ Department of Medical Microbiology, Faculty of Health Sciences University of Nairobi Nairobi Kenya; ^9^ Department of Forestry and Wildlife Management, School of Natural Resources, Environmental Studies and Agriculture Maasai Mara University Narok Kenya

**Keywords:** climate change, East Africa, ecological modeling, ensemble prediction, *Mops pumilus*

## Abstract

Synanthropic bats live in close proximity to humans and domestic animals, creating opportunities for potential pathogen spillover. We explored environmental correlates of occurrence for a widely distributed synanthropic African bat, *Mops pumilus—*a species associated with potential zoonotic viruses*—*and estimated current and future environmental suitability in the Taita Hills region and surrounding plains in Taita–Taveta County in southeast Kenya. To project future environmental suitability, we used four Coupled Model Intercomparison Project Phase 6 general circulation models that capture temperature and precipitation changes for East Africa. The models were parameterized with empirical capture data of *M*. *pumilus* collected from 2016 to 2023, combined with satellite‐based vegetation, topographic, and climatic data to identify responses to environmental factors. The strongest drivers for current environmental suitability for *M*. *pumilus* were short distance to rivers, higher precipitation during the driest months, sparse vegetation*—*often related to urban areas*—*and low yearly temperature variation. To predict current and future areas suitable for *M*. *pumilus*, we created ensemble niche models, which yielded excellent predictive accuracies. Current suitable environments were located southward from the central and southern Taita Hills and surrounding plains, overlapping with urban centers with the highest human population densities in the area. Future projections for 2050 indicated a moderate increase in suitability range in the southern portion of the region and surrounding plains in human‐dominated areas; however, projections for 2090 showed a slight contraction of environmental suitability for *M*. *pumilus*, potentially due to the negative impact of increased temperatures. These results show how environmental changes are likely to impact the human exposure risk of bat‐borne pathogens and could help public health officials develop strategies to prevent these risks in Taita–Taveta County, Kenya, and other parts of Africa.

## Graphical abstract

Bats are reservoirs for emerging pathogens and may pose significant risks to human health. Understanding spatial overlap between bats and humans across landscapes is a necessary requirement for developing exposure prevention strategies against bat‐borne zoonotic pathogens. We explored environmental correlates of occurrence for a widely distributed synanthropic African bat, *Mops pumilus*—a species associated with potential zoonotic viruses—and estimated current and future environmental suitability in Taita–Taveta County in southeast Kenya. Current suitable environments were located southwards from central Taita Hills overlapping with urban centers, and future predictions demonstrate how these areas will change—first, the localized range of *M*. *pumilus* will increase in human‐dominated areas by 2050, and thereafter, environmental suitability will decrease and become more fragmented indicating the potential negative impact of higher temperature increase on the species. The results show how environmental changes are likely to impact human exposure risk of bat‐borne pathogens and could help public health officials develop strategies to prevent these risks in Taita–Taveta County, Kenya, and other parts of Africa. Figure was created with Biorender.com.

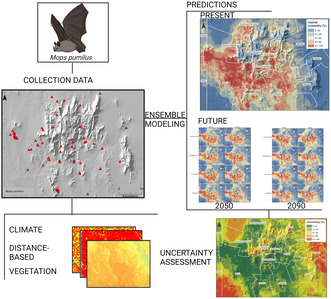

## 1 INTRODUCTION

Understanding the spatial overlap between humans and wildlife across landscapes is necessary for developing strategies that prevent human exposure to wildlife‐borne zoonotic pathogens. In anthropogenic spaces, synanthropic wildlife species live alongside humans and their domestic animals. Urban wildlife poses a greater risk of human exposure to pathogens because of high contact rates with humans and because of the variety of zoonotic pathogens that they harbor (Albery et al., 2022; Bradley & Altizer, 2007; Plowright et al., 2017).

The African continent has the world's fastest‐growing human population. Kenya ranks among the most populated nations in Africa with a total population of 52.5 million people in 2021, which is projected to double by the end of the 21st century (UN, 2022). With accelerating urbanization and agriculturalization to accommodate this population growth, increasing human interactions with wildlife are likely (Baker et al., 2022). Together with the impacts of climate change, developing infrastructure, and human mobility, these phenomena boost disease emergence and spillover risk across the landscape.

Bats are a diverse group of mammals that can be found in areas with varying degrees of urbanization and on all continents inhabited by humans (Simmons & Cirranello, 2023). Many bat species are synanthropic, with several species continuing to adapt to and exploit anthropogenic areas (Schoeman, 2016). The use of urban areas brings bats and humans into shared spaces where human–bat contact can occur (Russo & Ancillotto, 2015). Bats are reservoirs for emerging pathogens (Olival et al., 2017), including highly pathogenic viruses from families like Coronaviridae (Lane et al., 2022; Tong et al., 2009), Adenoviridae (Waruhiu et al., 2017), Paramyxoviridae (Lane et al., 2022), and Filoviridae (Amman et al., 2020; Forbes et al., 2019; Goldstein et al., 2018; Kareinen et al., 2020). Bats are additionally known to be infected by highly pathogenic species of Togaviridae and Flaviviridae (Calisher et al., 2006; Kading et al., 2022; Karan et al., 2019; Waruhiu et al., 2017). Because of their proximity to humans and their ability to host a diversity of pathogens, some synanthropic bat species may pose significant risks to human health, and intervention strategies are needed to understand the potential distribution of these species across landscapes.

The insectivorous little free‐tailed bat, *Mops pumilus* (family Molossidae) is widely distributed across the African continent; the geographical range of the species extends from the Horn of Africa (Ethiopia, Djibouti, Eritrea) to the Middle East (Yemen and Saudi Arabia), including areas identified as hotspots for emerging infectious diseases (Bett et al., 2020). The species is found in a diversity of environments, including woodland, rainforest, bushland, thicket, and agricultural areas but also in urban and suburban areas (Schoeman, 2016; Wilson & Mittermeier, 2019). *Mops pumilus* roosts communally in groups that can number from a few individuals to several thousand, often sharing roosts in human dwellings with several other synanthropic bat species (Jackson et al., 2024; Wilson & Mittermeier, 2019). Despite the wide geographical range of *M*. *pumilus* in Africa and its frequent interactions with humans and domestic animals (Jackson et al., 2024; Lunn et al., 2023), environmental factors that drive its environmental suitability have not been previously studied. Bat distribution is influenced by a variety of environmental and behavioral factors that impact their movement across the landscape. For example, precipitation and temperature directly impact environmental suitability for bats via their effects on flight activity and thermoregulation (Voigt et al., 2011). Vegetation density and structure, along with water presence, can regulate prey biomass (Ober & Hayes, 2008; Wolbert et al., 2014). Furthermore, while short‐term weather conditions are important predictors of bat environmental suitability, long‐term climatic conditions can explain local variations in bat distribution (Erickson & West, 2002).

Species distribution modeling can be used to identify landscape areas conducive to a particular wildlife species along with the elements that have the greatest impact on the environmental niche for that species (Guisan & Zimmermann, 2000). When applied to a potential zoonotic pathogen host like *M*. *pumilus*, species distribution modeling can inform human exposure risk over heterogeneous landscapes. In this study, we aim to (1) identify the most influential environmental variables driving the spatial occurrence of *M*. *pumilus*; (2) use this information to project the environmental suitability for *M*. *pumilus* across the county; and (3) project future distribution across the county under four climate projections for 2050 and 2090. This pre‐emptive study creates information that is critical for identifying areas of high overlap between this common synanthropic bat species and humans, which may represent regions with a high risk of human exposure to emerging zoonotic pathogens.

## 2 MATERIALS AND METHODS

### 2.1 Study area

**FIGURE 1:**
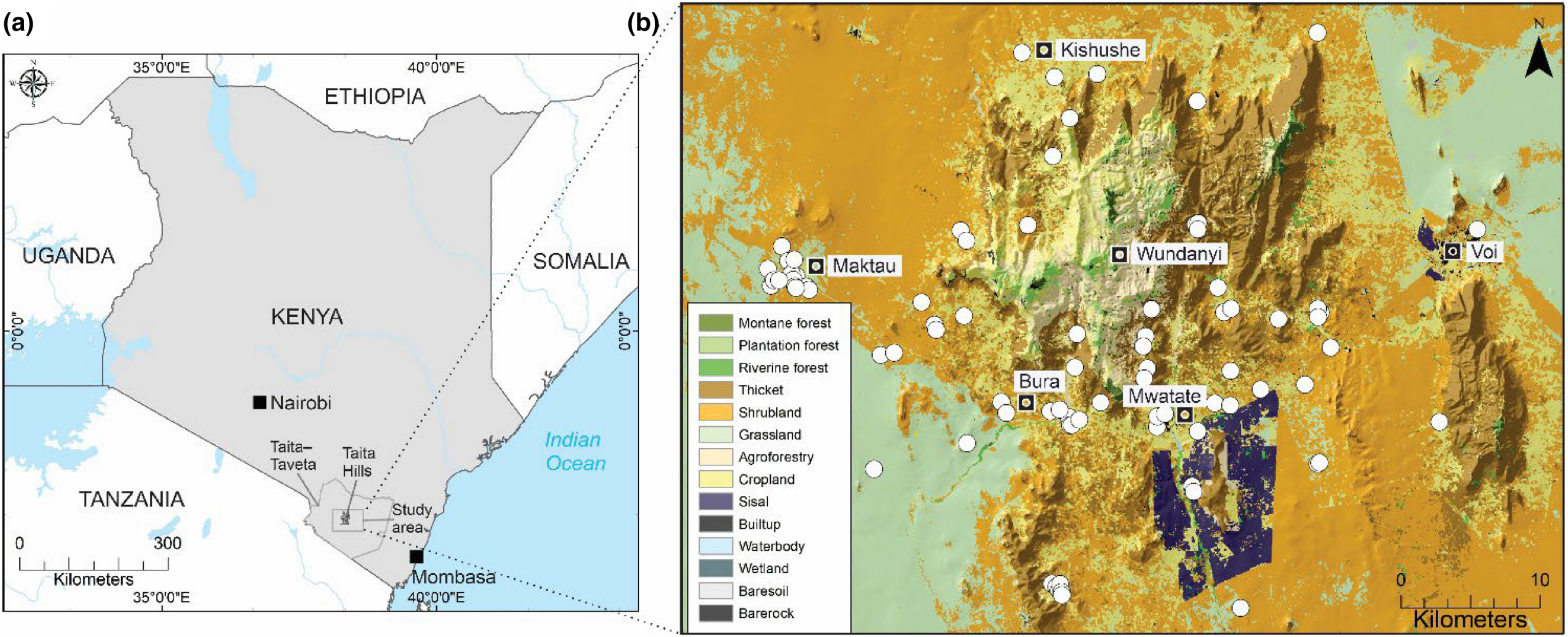
Study area in the Taita Hills and surrounding plains in Taita–Taveta County, southeast Kenya (a, b). Map indicating *Mops pumilus* occurrence data with 81 presence points across the study area and different vegetation types (b).

Our study was conducted in the Taita Hills and surrounding plains in Taita–Taveta County, southeast Kenya (Figure 1a,b). This area is recognized as a hotspot for emerging zoonotic disease risk (Allen et al., 2017). Previous work has identified coronaviruses in *M*. *pumilus* bats in the area (Waruhiu et al., 2017). The study area covers an approximate area of 2700 km^2^, including the hills (1000 km^2^) and surrounding plains. Taita–Taveta County is divided into 20 administrative districts (*wards), 15 of which extend to our study area. This area is characterized by high wildlife diversity and habitat heterogeneity; the hills have cloud forest from 1400 to 2200 m surrounded by lower‐elevation (400–1400 m) grassland, woodland, semiarid shrubland, and dry savanna (Abera et al., 2022; Platts et al., 2011). The climate is semiarid, with an average annual temperature of 23°C (Autio et al., 2021; Ogallo et al., 2019). Typically, there are two rainy seasons—March to May/June and October to December—with an average annual rainfall of 150–600 mm in the lowlands and 800–1200 mm in the highlands (Autio et al., 2021; Ogallo et al., 2019). Urbanization has dramatically increased during recent decades in Taita–Taveta County, with a 700% increase in developed landcover during the past decade and a steadily increasing human population (Kenya National Bureau of Statistics, 2019; Nyongesa et al., 2022). Human–wildlife interactions have increased because of higher rates of environmental loss and forest degradation brought on by altered agricultural activity, accelerated climate change, and a rapidly expanding human population (Maeda, 2012; Munyao et al., 2020).

### 2.2 Bat occurrence data

Occurrence data included a total of 84 presence locations for *M*. *pumilus* recorded between 2016 and 2023. Most *M*. *pumilus* were captured from houses (*N* = 79), with a few captured flying over waterbodies (*N* = 5). For bat trapping, we used single‐, double‐, and triple‐high mist nets and hand nets in buildings and at natural flyways over water sources to capture bats (Lunn et al., 2023). Buildings that were used by bats were identified through house‐to‐house surveys and community conversations (Jackson et al., 2024). Captured bats were identified to species level in the field using existing keys for bats in East Africa (Patterson & Webala, 2012).

### 2.3 Predictors of bat environmental suitability

We incorporated several environmental predictors into models based on their known or suspected influence on bat distributions (Cooper‐Bohannon et al., 2016; Koch et al., 2020; Pigott et al., 2014; Reed Hranac et al., 2019). Environmental data for the study area included precipitation, temperature, topographic, vegetation, and distance‐based variables obtained from satellite imagery, GIS layers, and interpolated data (Table S1). As some environmental data had a higher resolution (20 m) than the other layers, we downscaled bioclimatic data (~1000 m) acquired from the WorldClim database (version 2.1; Fick & Hijmans, 2017). We used geographic weighted regression for grid downscaling in QGIS (version 3.28.4) with the Saga Next Generation plug‐in to downscale (100 m resolution) the environmental data by using the digital elevation model. We also incorporated 19 bioclimatic variables into the models to compare current and future environmental suitability for *M*. *pumilus*.

To predict future environmental suitability for *M*. *pumilus*, we used the following four CMIP6 general circulation models (GCMs): EC‐Earth3‐Veg, HadGEM3‐GC31‐LL, IPSL‐CM6A‐LR, and MRI‐ESM2‐0. We selected these GCMs, as they vary in climate sensitivity (Lange, 2021) and can capture extreme seasonal precipitation indices, particularly in East Africa (Akinsanola et al., 2021). In conjunction with each GCM scenario, we used two shared socioeconomic pathways (SSPs): 2.45 (medium change) and 5.85 (high change). The SSP2.45 scenario represents the medium pathway for future greenhouse gas emissions, with a temperature rise of 3°C. It follows historical growth trends in development and reduced fossil‐fuel dependence, global population growth is moderate, and environmental systems are facing certain degradation (Riahi et al., 2017; Tebaldi et al., 2021). The SSP5.85 scenario represents the upper boundary of future predictions, with a temperature rise of 5°C by 2100. It is based on socioeconomic progress, reduced global inequality, a growing world economy, strong reliance on fossil fuels, and intensive development and energy consumption (Riahi et al., 2017; Tebaldi et al., 2021). Lastly, future environmental suitability for *M*. *pumilus* was predicted based on bioclimatic data for 2041–2060 and 2081–2100 using the mean value of the climate variable for each period (2050 and 2090).

### 2.4 Data preparation and analysis

To reduce spatial autocorrelation, bat occurrence data were spatially thinned using R package *Wallace* (Kass et al., 2018) with the *spThin* approach. With spatial autocorrelation, data or residuals are correlated with themselves rather than being independent (Drew et al., 2011) and may inflate the effective sample size and bias parameter estimates. For *M*. *pumilus* observation data, we used a spatial thinning buffer of 100 m to incorporate the highest possible number of presences. After data thinning, *M*. *pumilus* data consisted of 81 presence points (Figure 1b). To model the environmental niche, we generated three pseudoabsence points per presence point via the *random* strategy (*N* = 243) across 10 replication sets, as recommended (Barbet‐Massin et al., 2012; Thuiller et al., 2023). In the final models, presence and pseudoabsence points were equally weighted (Barbet‐Massin et al., 2012). We used the *biomod2* platform in R (version 3.4.6; Thuiller et al., 2023) to create species distribution models (SDMs) to identify areas with suitable environmental conditions for *M*. *pumilus*.

All geospatial datasets, including environmental and bioclimatic data, were processed in Esri ArcGIS (version 10.8; Environmental Systems Research Institute (ESRI), 2023) or QGIS (version 3.28.4) and were set to the same spatial extent, geographic coordinate system (Arc 1960 UTM Zone 37S, EPSG:21037), and resolution (100 × 100 m). Multicollinearity of the variables was investigated using variance inflation factors (VIFs), as implemented in R package *usdm* (Belsley et al., 1980; Naimi, 2017). Correlated variables were excluded in a stepwise procedure using a commonly applied threshold value of 10 (Chatterjee & Hadi, 2013; Sulaiman et al., 2019); 9 out of 24 variables were included in the final modeling analysis to predict current environmental suitability for *M*. *pumilus* (Table S1). For current and future projections including only climatic data, 6 out of 19 bioclimatic variables were included in the final analysis after reducing multicollinearity (Table S1).

The following eight predictive modeling techniques were employed in our ensemble approach: generalized linear model (GLM) (McCullagh, 1989), generalized additive model (GAM) (Hastie, 1990), classification tree analysis (CTA) (Breiman, 1984), artificial neural networks (ANN) (Ripley, 1996), multivariate adaptive regression splines (MARS) (Friedman, 1991), generalized boosting model (GBM) (Ridgeway, 1999), random forest (RF) (Breiman, 2001), and maximum entropy (MAXNET) (Phillips et al., 2017). Flexible discriminant analysis (FDA) and surface range envelope (SRE) were excluded because of generally poor predictive performance (Elith et al., 2006; Zhao & Gao, 2015). The models were run using the default settings of *biomod2* (Thuiller et al., 2023). We used a cross‐validation technique in which the thinned dataset was divided into two parts, one to calibrate the models (70%) and another to evaluate them (30%) (Guisan & Zimmermann, 2000). We repeated the calibration and evaluation sets 10 times for each model and pseudoabsence dataset (800 model evaluation runs in total). To reduce uncertainty related to the choice of a single modeling technique, we built ensemble predictions using the ensemble mean method (Araújo & New, 2007). This approach produces the ensemble prediction by averaging predictions across the best‐performing individual models (0.7 < area under the curve, AUC < 1.0) (Thuiller et al., 2023). Predictions based on ensemble mean models were used as an input for environmental suitability maps of *M*. *pumilus*. The current suitability distribution result, including only climatic data (Table S1), was further projected to predict the species' future distributions under previously mentioned GCMs (see section 2.3 Predictors of Bat Environmental Suitability).

### 2.5 Accuracy assessment

Sensitivity (the proportion of correctly predicted presences) and specificity (the proportion of correctly predicted pseudoabsences) were calculated to quantify omission errors (Fielding & Bell, 1997). AUC and true skill statistics (TSS) (Allouche et al., 2006) were used to measure model ability to distinguish between presence and pseudoabsence classes. AUC scores range from 0 to 1, with 0.5 being the threshold for predictions better than random (Fielding & Bell, 1997), and >0.7 being an acceptable threshold for predictions (Morán‐Ordóñez et al., 2017). TSS scores range from −1 to 1, where 1 indicates a perfect ability to distinguish suitable habitats from unsuitable ones, while values of zero or less indicate a performance no better than random (Allouche et al., 2006). Variable importance, referred to here as the relative percent contribution of a predictor to model outputs, was extracted from the *biomod2* output, with higher values indicating higher influence on the ensemble mean model (Thuiller et al., 2023). Partial dependency plots were generated showing the average effect of each covariate on the overall response. To estimate current and future environmental suitability for *M*. *pumilus* based on climate data, we only present variable importance and partial dependence plots for current suitability, as these variables mainly followed a similar importance order in all predicted future scenarios (*N* = 16). To detect changes of suitable areas for *M*. *pumilus* between current and future projections, we first classified areas as suitable or unsuitable based on threshold values that maximized sensitivity and specificity in each projection. We then calculated the percentage of suitable habitats for each projection and compared them to determine changes between present, 2050, and 2090 projections. Suitability maps were first created using R software and were afterwards modified in ArcGIS.

## 3 RESULTS

### 3.1 Model performance

The generated ensemble niche models for estimating current environmental suitability for *M*. *pumilus* performed strongly (AUC = 0.95 and TSS = 0.75; Table S2). The mean predictive performance of all 16 future scenarios was 0.93 based on AUC (range = 0.92–0.94) and 0.72 based on TSS (range = 0.69–0.76). Of the individual models used to create an ensemble niche model, the RF, GBM, and GAM models had the strongest performance (Figure S1). The ensemble mean model identified unsuitable environments better than suitable environments in the current predictions (sensitivity = 80.3%, specificity = 94.7%; Table S2). For future predictions, mean sensitivity based on AUC was 83.7% (range = 76.5–91.4%) and specificity was 88.3% (range = 82.1–91.8%).

### 3.2 Predictor contributions

**FIGURE 2:**
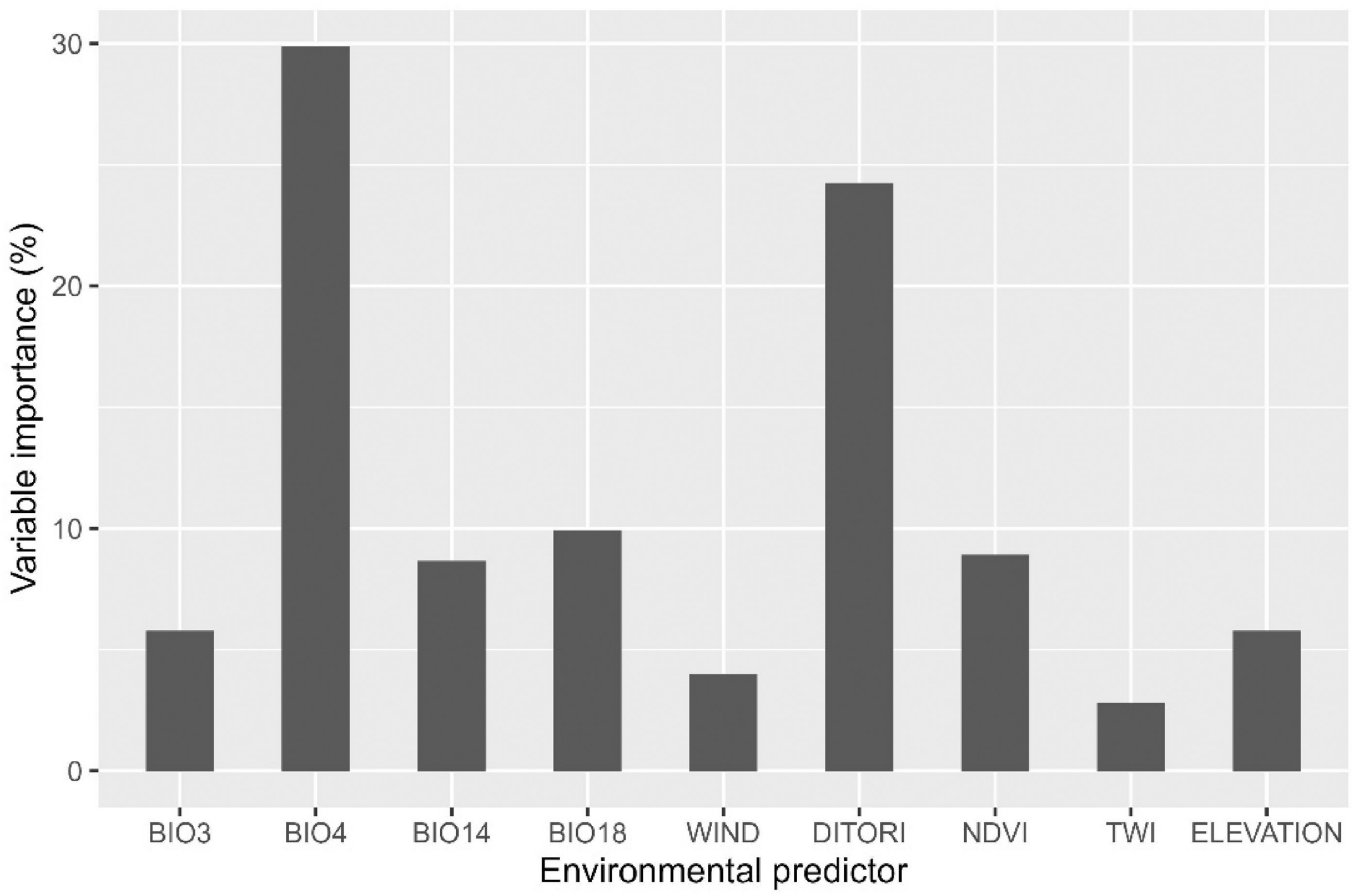
Relative contributions of environmental variables for estimating current environmental suitability for *Mops pumilus* by the ensemble mean model. BIO14, precipitation of the driest month; BIO18, precipitation of the warmest quarter; BIO3, isothermality; BIO4, temperature seasonality; DITORI, distance to river; NDVI, normalized difference vegetation index; TWI, topographic wetness index.

**FIGURE 3:**
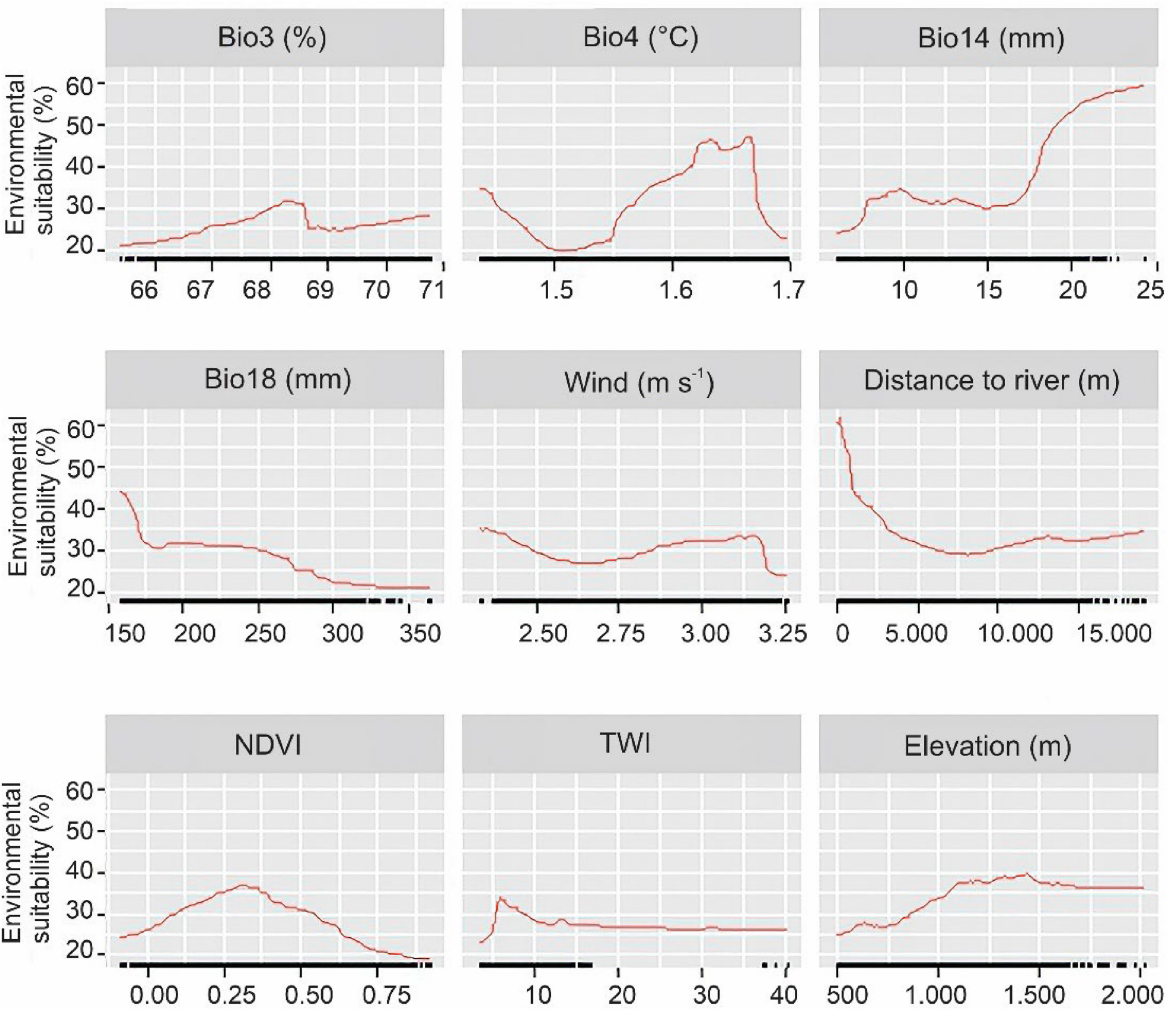
Partial dependency plots for estimating current environmental suitability for *Mops pumilus* produced by the ensemble mean model. BIO14, precipitation of the driest month; BIO18, precipitation of the warmest quarter; BIO3, isothermality; BIO4, temperature seasonality; NDVI, normalized difference vegetation index; TWI, topographic wetness index.

Our models showed that the environmental suitability of *M*. *pumilus* was influenced by several environmental and bioclimatic variables (Figures 2 and 3). The highest relative contributions were by BIO4 = temperature seasonality (29.9%) followed by distance to river (24.2%), BIO18 = precipitation of warmest quarter (9.9%), normalized difference vegetation index, NDVI (8.9%), BIO14 = precipitation of driest month (8.7%), elevation (5.8%), BIO3 = isothermality (5.8%), and wind speed (4.0%). Topographic wetness index (TWI, 2.8%) was the least important predictor among the models.

Locations with relatively low temperature variation within a year (1.55–1.66°C), high precipitation during the driest month (>16 mm), low levels of temperature variability within an average month relative to the year (67–68.5%), a high TWI (>4), sparse vegetation (0.1 < NDVI < 0.3), and elevations between 900 and 1500 m had higher environmental suitability for *M*. *pumilus* (Figure 3). Longer distance to rivers (>500 m), high precipitation during the warmest quarter (>160 mm), and high wind speed (>1.3 m s^−1^) were negatively associated with *M*. *pumilus* suitability (Figure 3).

### 3.3 Current environmental suitability for *Mops pumilus*

**FIGURE 4:**
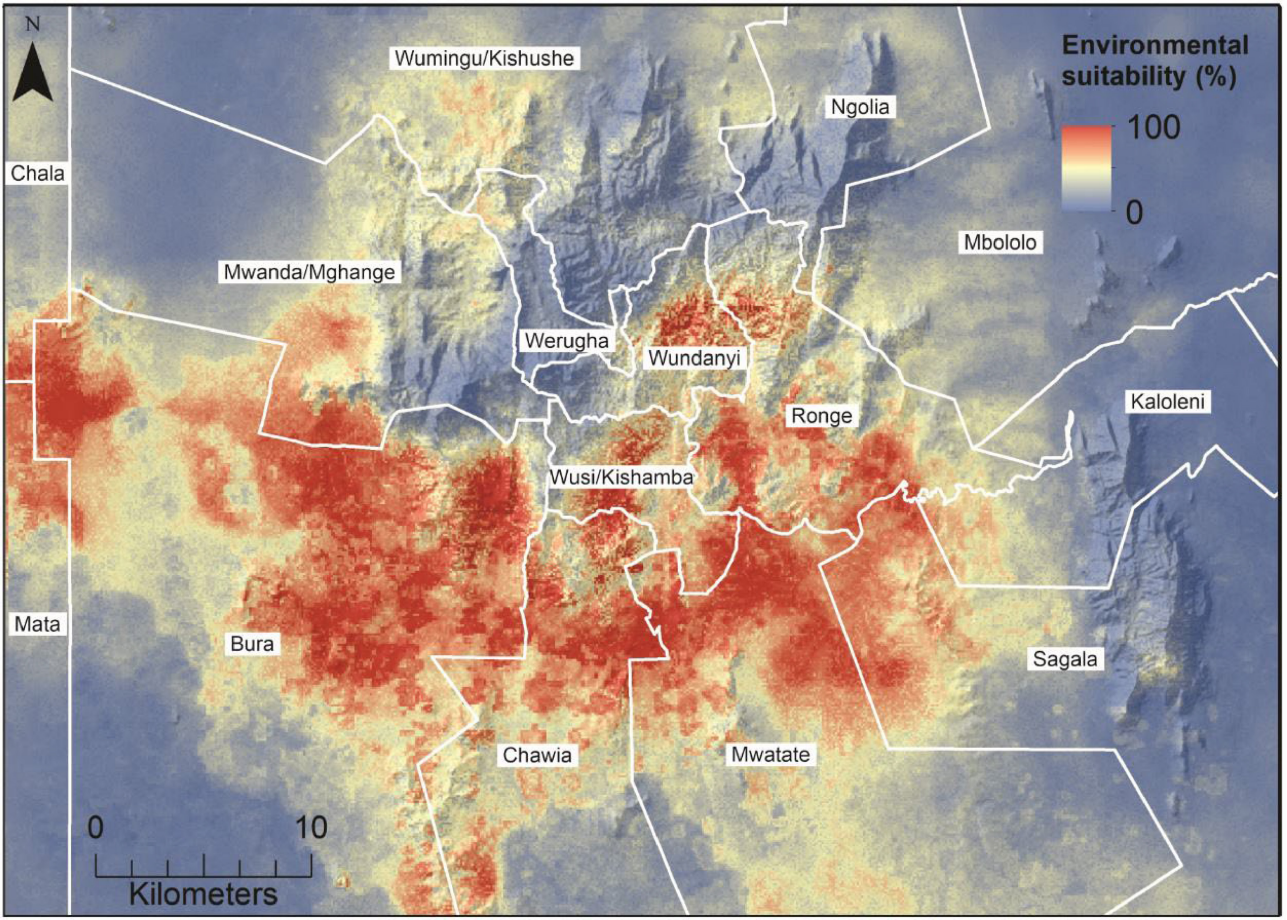
Current environmental suitability for *Mops pumilus* presented by administrative ward in the study area in Taita–Taveta County by the ensemble mean method over several modeling methods.

Our models estimate high levels of environmental suitability for *M*. *pumilus* in current environmental settings across large areas in the Taita Hills and surrounding plains in Taita–Taveta County (Figure 4). Areas with highest suitability for *M*. *pumilus* were found in 12 of the 15 wards located in the study area, with highest suitability predicted in savanna, grassland, shrubland, and developed low‐elevation regions. Areas at elevations lower than 750 m and greater than 1500 m or areas with minimal development were estimated to have low to moderate suitability for *M*. *pumilus*.

### 3.4 Future environmental suitability for *Mops pumilus*

**FIGURE 5:**
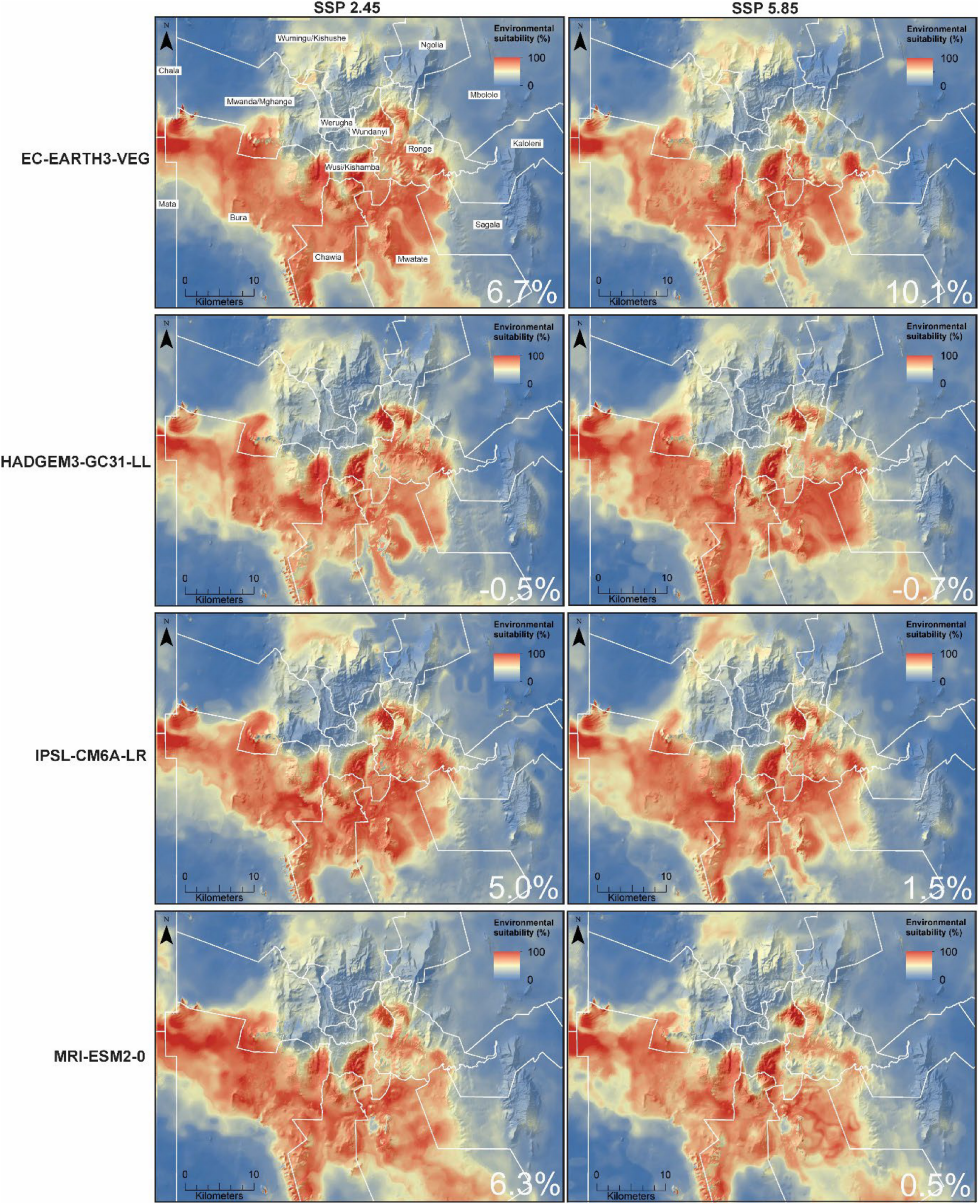
Future environmental suitability for *Mops pumilus* in the Taita Hills and surrounding plains projected for 2050 using four general circulation models (GCMs) and two shared socioeconomic pathways (SSPs) based on the ensemble mean method over several modeling methods. The percentage change in suitable habitat between the present and future (2050) is indicated in the lower right corner of each panel.

Our models predicted changes in the environmental suitability for *M*. *pumilus* in the Taita Hills and surrounding plains by 2050 (Figure 5). Increases in environmental suitability are likely across the study area, especially in mid‐ to high‐elevation areas and in undisturbed savanna, shrubland, and woodland in the southern reaches of the Taita Hills and surrounding plains. Our models showed minimal contraction (−0.7%) or no contraction of environmental suitability in any administrative ward by 2050. The EC‐Earth3‐Veg and MRI‐ESM2‐0 models with SSP2.45 scenarios yielded the largest increases in environmental suitability (6.3–10.1%) for *M*. *pumilus* by 2050.

**FIGURE 6:**
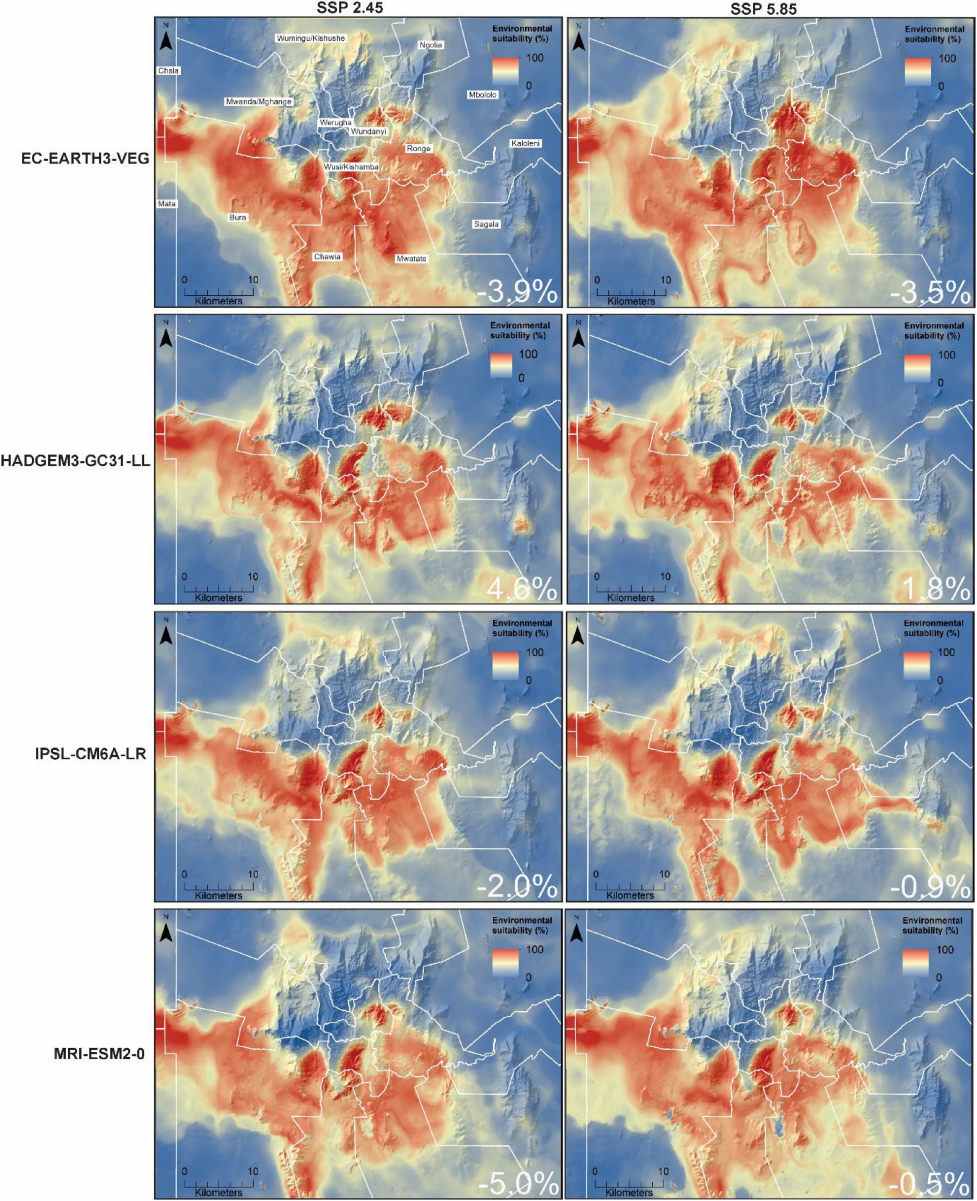
Future environmental suitability for *Mops pumilus* in the Taita Hills and surrounding plains projected for 2090 using four general circulation models (GCMs) and two shared socioeconomic pathways (SSPs) based on the ensemble mean method over several modeling methods. The percentage change in suitable habitat between 2050 and 2090 is indicated in the lower right corner of each panel.

In contrast, the considered GCMs, excluding HadGEM3‐GC31‐LL, predicted a slightly contracting distribution (−0.5–5.0%) for *M*. *pumilus* in all administrative wards between 2050 and 2090 (Figures 5 and 6). The EC‐Earth3‐Veg, IPSL‐CM6A‐LR, and MRI‐ESM2‐0 models, with low and medium change scenarios, predicted suitability in low‐elevation savanna, shrubland, and woodland to decrease substantially, with high environmental suitability for *M*. *pumilus* largely concentrated in mid‐to high‐elevation areas.

## 4 DISCUSSION

Here, for the first time, we developed SDMs to determine the drivers for environmental suitability for *M*. *pumilus*, to identify hotspot areas and to model changes in suitable habitats under future scenarios. Our projections indicate that *M*. *pumilus* inhabits large portions of the Taita Hills and surrounding plains, including hotspot areas of suitability coinciding with human development and agriculture. Future predictions demonstrate how these areas will change in the study area—first, the localized range of *M*. *pumilus* will increase in the short term, by 2050, but thereafter it will decrease and become more fragmented when the species' range contracts—as expected in the long term.

Our study shows that the current environmental suitability for *M*. *pumilu*s was associated with temperature, precipitation, and topographic variables, vegetation cover, and waterway presence, which is mainly congruent with studies of other bat species (Koch et al., 2020; Lee et al., 2012). The results indicate that *M*. *pumilus* is sensitive to changes in temperature seasonality. High temperature variation throughout the year has been found to impact the basal metabolic rate of other bat species (Downs et al., 2012). Additionally, extremely high or low temperatures over the course of a year may affect the physiological tolerance of the species, for example by lowering net primary productivity and thereby reducing the availability of insect prey (Schloss et al., 1999; Vinson & Hawkins, 2003). Our findings also demonstrate that locations with shorter distances to rivers and higher precipitation during the driest month were associated with higher suitability for *M*. *pumilus*. Water is a limiting resource in semiarid climates like that of southeast Kenya, and bats may use waterbodies, such as rivers, for hydration (Katunzi et al., 2021; Rainho & Palmeirim, 2011). The presence of waterbodies and higher precipitation also increase insect biomass, which is crucial for bat reproduction (Nurul‐Ain et al., 2017). Our findings are also consistent with results from previous studies in other African countries that identified temperature and precipitation variables as key drivers of bat habitat suitability for other bat species and families (Arumoogum et al., 2019; Cooper‐Bohannon et al., 2016; Schoeman et al., 2013). However, excessively high precipitation levels increase their flight metabolism, which in turn decreases their body mass (Davy et al., 2022). Additionally, locations with high suitability for *M*. *pumilus* were associated with sparse vegetation. This is evident, as synanthropic bats roost in buildings, particularly in urban environments with sparse vegetation cover.

The projected suitability for *M*. *pumilus* was highest at low‐ to mid‐elevation areas just south of the highest mountains of the Taita Hills. These areas are dominated by moderate rates of human development and agriculture containing several of the county's urban centers (Ojwang’ et al., 2017). Human population growth in Taita–Taveta County is steadily increasing (Kenya National Bureau of Statistics, 2019), and rapid urbanization creates more buildings, that is, roost sites for bats. Although high suitability areas for *M*. *pumilus* are mostly located in low‐ to mid‐elevation areas, environmental conditions in higher‐elevation areas in Taita–Taveta County may not be a limiting factor for the species, as our findings indicate that the suitability for *M*. *pumilus* remains high at elevations greater than 1500 m. This finding is somewhat contradictory to earlier studies in which *M*. *pumilus* has not been captured at elevations above 1400 m (Benda et al., 2019; Katunzi et al., 2021; Lane et al., 2022).

Future predictions show that human‐dominated areas will continue to be acceptable for use by this synanthropic bat species in the near future, although this trend varies with time. The immediate projected range expansion may be correlated with predicted increases in temperatures and precipitation, and the plasticity of the thermal tolerance of *M*. *pumilus* may be beneficial as the climate changes in this region (Marsden et al., 2022). However, subsequent prediction scenarios for 2090 indicate a slight contraction of the suitability of the southern study area, where the environmental suitability for *M*. *pumilus* was previously highest. In our study, suitability for *M*. *pumilus* was negatively associated with temperature extremes, suggesting that the species could probably be impacted by global warming, as its suitable habitats would be reduced. At 1.5°C, 2°C, and 3°C of global warming above preindustrial levels, mean annual temperatures in East Africa are estimated to average 0.6–2.1°C warmer than the 1994–2005 average (IPCC, 2023). This temperature increase may be too extreme for *M*. *pumilus* to tolerate physiologically and may have negative impacts on the species' insect prey (Erickson & West, 2002). Therefore, the risk of human contact with *M*. *pumilus* may increase in the near future but will likely decrease as the severity of climate change increases.

While our models had strong predictive performance, there are some limitations to the interpretation of our data. Although CMIP6 models depict improved performance in the climate simulations relative to earlier CMIP5 models (Ayugi et al., 2021), any long‐term future projections are always subject to a range of assumptions and limitations. We have endeavored to address this by using ensemble mean models, four GCMs known to capture specific features of East African climate with two SSPs and 800 suitability model runs (Akinsanola et al., 2021). The future projections for *M*. *pumilus* suitability are meant to show average trends and should not be understood as being predictive for specific years. High model uncertainty in the southwestern, southeastern, and central parts of the study area may be due to sampling bias, as sampling focused on building roosts and could not be conducted in several of the protected areas.

Here, we investigated the drivers of occurrence for *M*. *pumilus* and identified environmentally suitable habitats for the species under current and future scenarios. We studied these aspects in the Taita Hills and surrounding plains in southeastern Kenya, but the results are transferable to other regions in Africa that are not far from their geographical distance or from the core of a species' range and to regions with topographical variation (Rousseau & Betts, 2022). Here, we used high‐quality occurrence data of *M*. *pumilus* instead of aggregated observations from big data repositories that may often be prone to spatial bias (Beck et al., 2013). Our findings may help to identify areas where potential exposure to bat‐borne pathogens may occur and potentially allow a better estimation of where precautionary steps and preventive actions may become necessary in the future.

## 5 SUMMARY AND CONCLUSIONS

We have identified environmental drivers, current environmental suitability, and possible future scenarios for *M*. *pumilus* by utilizing empirical data from the Taita Hills and surrounding plains in Kenya. The focus area is representative of much of rural sub‐Saharan Africa, and this is a common and widely distributed bat species that is host to virus groups with public health implications. Based on the results, we found that low variation in temperature within a year, short distance to rivers, sparse vegetation, and higher precipitation during the driest month drive the strongest environmental suitability for *M*. *pumilus*. Predicted current environmental suitability indicated greatest suitability in low‐ to mid‐elevation areas south of the highest mountains in the Taita Hills. These areas involve urban centers with the highest human population densities in the area and are located in the vicinity of rangeland and wildlife conservation areas. Most of the human‐dominated areas are predicted to remain suitable for *M*. *pumilus* in the near future but to shrink slightly towards the end of the century. Our results may have considerable public health value not only in Taita–Taveta County but also in other parts of Africa with comparable environmental conditions. Furthermore, our findings can be used to better estimate the locations where preventive measures will be required and to identify potential exposure sites to bat‐borne pathogens in a landscape known for its risk of zoonotic disease emergence.

## AUTHOR CONTRIBUTIONS

**Ruut J. Uusitalo:** Conceptualization (equal); data curation (equal); formal analysis (equal); funding acquisition (equal); investigation (equal); methodology (equal); project administration (equal); resources (equal); software (equal); supervision (equal); validation (equal); visualization (equal); writing – original draft (equal); writing – review and editing (equal). **Reilly T. Jackson:** Conceptualization (equal); data curation (equal); writing – original draft (equal); writing – review and editing (equal). **Tamika J. Lunn:** Conceptualization (equal); data curation (equal); writing – review and editing (equal). **Essi M. Korhonen:** Funding acquisition (equal); project administration (equal); writing – original draft (equal); writing – review and editing (equal). **Joseph G. Ogola:** Data curation (equal); writing – review and editing (equal). **Paul W. Webala:** Data curation (equal); writing – review and editing (equal). **Tarja A. Sironen:** Funding acquisition (equal); project administration (equal); resources (equal); supervision (equal); writing – review and editing (equal). **Kristian M. Forbes:** Data curation (equal); project administration (equal); resources (equal); supervision (equal); writing – original draft (equal); writing – review and editing (equal).

## FUNDING INFORMATION

R.U. was supported by the Sakari Alhopuro Foundation. This research was supported by the Academy of Finland through the DEVELOP project (decision No #1339510), the Maj and Tor Nessling Foundation, and the Arkansas Biosciences Institute.

## CONFLICT OF INTEREST STATEMENT

The authors declare no conflict of interest.

## Supporting information


Data S1:

## Data Availability

Data and scripts used in the analyses are available from the Dryad Digital Repository: https://datadryad.org/stash/share/2wkMjd‐yN06NI‐zTdE0mhPaCApn7bF2PJ8FUL6yF2Io.
