# Supplementary material for: Current and future environmental suitability for bats hosting potential zoonotic pathogens in rural Kenya
Source: Ecol Evol. 2024 Jun 14;14(6):e11572. doi: 10.1002/ece3.11572 (PMC11177178; doi:10.1002/ece3.11572)
Supplement: Supplementary file 1 — Data S1: [file ECE3-14-e11572-s001.docx]

Table S1. Environmental and climatic data incorporated into models examining environmental suitability for the synanthropic bat species *Mops pumilus* in the Taita Hills and surrounding plains, Taita–Taveta County, southeast Kenya.

| **Data layer(s)** | **Unit** | **Year** | **Spatial resolution (m)** | | **In current prediction (+/−**) | **In current prediction with only climate data (+/−**) | **In future predictions with only climate data (+/−**) | **Modifications** | **References** |
| --- | --- | --- | --- | --- | --- | --- | --- | --- | --- |
| Elevation | m | 2005 | | 20 | + | **−** | **−** | Obtained from digital elevation model (DEM) | Clark & Pellikka, 2005 |
| Topographic wetness index | **−** | 2005 | | 30 | + | **−** | **−** | Downscaled in QGIS to 100 m resolution using DEM | Clark & Pellikka, 2005 |
| Normalized difference vegetation index | **−** | 2015 | | 30 | + | **−** | **−** | Downscaled in QGIS to 100m resolution using DEM | Wolters et al., 2018 |
| Wind | m s**^−^**^1^ | Average climate data for 1970–2000 | | ~1000 | + | **−** | **−** | Downscaled in QGIS to 100m resolution using DEM | Fick & Hijmans, 2017. Available online: https://www.worldclim.org/data/index.html. |
| Distance to river | m | **−** | | 20 | + | **−** | **−** | Euclidean distance to river was calculated in ArcGIS | **−** |
| BIO1 = Annual mean temperature | °C | Average climate data for 1970–2000 | | ~1000 | **−** | **−** | **−** | Downscaled in QGIS to 100m resolution using DEM | Fick & Hijmans, 2017. Available online: https://www.worldclim.org/data/index.html. |
| BIO2 = Mean diurnal range | °C | Average climate data for 1970–2000 | | ~1000 | **−** | **−** | **−** | Downscaled in QGIS to 100m resolution using DEM | Fick & Hijmans, 2017. Available online: https://www.worldclim.org/data/index.html. |
| BIO3 = Isothermality | °C | Average climate data for 1970–2000 | | ~1000 | + | + | + | Downscaled in QGIS to 100m resolution using DEM | Fick & Hijmans, 2017. Available online: https://www.worldclim.org/data/index.html. |
| BIO4 = Temperature seasonality | °C | Average climate data for 1970–2000 | | ~1000 | + | + | + | Downscaled in QGIS to 100m resolution using DEM | Fick & Hijmans, 2017. Available online: https://www.worldclim.org/data/index.html. |
| BIO5 = Max temperature of warmest month | °C | Average climate data for 1970–2000 | | ~1000 | **−** | **−** | **−** | Downscaled in QGIS to 100m resolution using DEM | Fick & Hijmans, 2017. Available online: https://www.worldclim.org/data/index.html. |
| BIO6 = Min temperature of coldest month | °C | Average climate data for 1970–2000 | | ~1000 | **−** | **−** | **−** | Downscaled in QGIS to 100m resolution using DEM | Fick & Hijmans, 2017. Available online: https://www.worldclim.org/data/index.html. |
| BIO7 = Temperature annual range | °C | Average climate data for 1970–2000 | | ~1000 | **−** | **−** | **−** | Downscaled in QGIS to 100m resolution using DEM | Fick & Hijmans, 2017. Available online: https://www.worldclim.org/data/index.html. |
| BIO8 = Mean temperature of wettest quarter | °C | Average climate data for 1970–2000 | | ~1000 | **−** | **−** | **−** | Downscaled in QGIS to 100m resolution using DEM | Fick & Hijmans, 2017. Available online: https://www.worldclim.org/data/index.html. |
| BIO9 = Mean temperature of driest quarter | °C | Average climate data for 1970–2000 | | ~1000 | **−** | **−** | **−** | Downscaled in QGIS to 100m resolution using DEM | Fick & Hijmans, 2017. Available online: https://www.worldclim.org/data/index.html. |
| BIO10 = Mean temperature of warmest quarter | °C | Average climate data for 1970–2000 | | ~1000 | **−** | **−** | **−** | Downscaled in QGIS to 100m resolution using DEM | Fick & Hijmans, 2017. Available online: https://www.worldclim.org/data/index.html. |
| BIO11 = Mean temperature of coldest quarter | °C | Average climate data for 1970–2000 | | ~1000 | **−** | **−** | **−** | Downscaled in QGIS to 100m resolution using DEM | Fick & Hijmans, 2017. Available online: https://www.worldclim.org/data/index.html. |
| BIO12 = Annual precipitation | mm | Average climate data for 1970–2000 | | ~1000 | **−** | **−** | **−** | Downscaled in QGIS to 100m resolution using DEM | Fick & Hijmans, 2017. Available online: https://www.worldclim.org/data/index.html. |
| BIO13 = Precipitation of wettest month | mm | Average climate data for 1970–2000 | | ~1000 | **−** | **−** | **−** | Downscaled in QGIS to 100m resolution using DEM | Fick & Hijmans, 2017. Available online: https://www.worldclim.org/data/index.html. |
| BIO14 = Precipitation of driest month | mm | Average climate data for 1970–2000 | | ~1000 | + | + | + | Downscaled in QGIS to 100m resolution using DEM | Fick & Hijmans, 2017. Available online: https://www.worldclim.org/data/index.html. |
| BIO15 = Precipitation seasonality | mm | Average climate data for 1970–2000 | | ~1000 | **−** | + | + | Downscaled in QGIS to 100m resolution using DEM | Fick & Hijmans, 2017. Available online: https://www.worldclim.org/data/index.html. |
| BIO16 = Precipitation of wettest quarter | mm | Average climate data for 1970–2000 | | ~1000 | **−** | + | + | Downscaled in QGIS to 100m resolution using DEM | Fick & Hijmans, 2017. Available online: https://www.worldclim.org/data/index.html. |
| BIO17 = Precipitation of driest quarter | mm | Average climate data for 1970–2000 | | ~1000 | **−** | **−** | **−** | Downscaled in QGIS to 100m resolution using DEM | Fick & Hijmans, 2017. Available online: https://www.worldclim.org/data/index.html. |
| BIO18 = Precipitation of warmest quarter | mm | Average climate data for 1970–2000 | | ~1000 | + | + | + | Downscaled in QGIS to 100m resolution using DEM | Fick & Hijmans, 2017. Available online: https://www.worldclim.org/data/index.html. |
| BIO19 = Precipitation of coldest quarter | mm | Average climate data for 1970–2000 | | ~1000 | **−** | **−** | **−** | Downscaled in QGIS to 100m resolution using DEM | Fick & Hijmans, 2017. Available online: https://www.worldclim.org/data/index.html. |

Table S2. Predictive performance of ensemble models estimating current and future environmental suitability for *M. pumilus*.

| **Projection** | **Socioeconomic pathway (SSP)** | **AUC** | **TSS** | **Sensitivity (by AUC)** | **Specificity (by AUC)** | **Sensitivity (by TSS)** | **Specificity (by TSS)** |
| --- | --- | --- | --- | --- | --- | --- | --- |
| **Current** | - | 0.95 | 0.75 | 80.25 | 94.72 | 80.25 | 94.51 |
| **Future (by 2050):** |  |  |  |  |  |  |  |
| EC-Earth3-Veg | 2.45 | 0.93 | 0.69 | 85.19 | 84.65 | 85.19 | 83.79 |
| EC-Earth3-Veg | 5.85 | 0.94 | 0.74 | 91.36 | 82.15 | 91.36 | 82.15 |
| HadGEM3-GC31-LL | 2.45 | 0.94 | 0.76 | 83.95 | 91.83 | 83.95 | 91.63 |
| HadGEM3-GC31-LL | 5.85 | 0.92 | 0.69 | 81.48 | 88.00 | 77.78 | 90.90 |
| IPSL-CM6A-LR | 2.45 | 0.93 | 0.71 | 83.95 | 86.84 | 83.95 | 86.80 |
| IPSL-CM6A-LR | 5.85 | 0.94 | 0.74 | 83.95 | 89.79 | 83.95 | 89.75 |
| MRI-ESM2-0 | 2.45 | 0.92 | 0.70 | 83.95 | 86.39 | 83.95 | 85.49 |
| MRI-ESM2-0 | 5.85 | 0.93 | 0.71 | 80.25 | 91.49 | 80.25 | 90.91 |
| **Future (by 2090):** |  |  |  |  |  |  |  |
| EC-Earth3-Veg | 2.45 | 0.93 | 0.69 | 80.25 | 89.73 | 80.25 | 88.90 |
| EC-Earth3-Veg | 5.85 | 0.92 | 0.71 | 86.42 | 84.31 | 86.42 | 84.19 |
| HadGEM3-GC31-LL | 2.45 | 0.94 | 0.74 | 83.95 | 89.79 | 83.95 | 89.75 |
| HadGEM3-GC31-LL | 5.85 | 0.94 | 0.74 | 83.95 | 90.10 | 83.95 | 89.93 |
| IPSL-CM6A-LR | 2.45 | 0.94 | 0.73 | 83.95 | 89.15 | 83.95 | 88.57 |
| IPSL-CM6A-LR | 5.85 | 0.94 | 0.76 | 86.42 | 89.87 | 86.42 | 89.74 |
| MRI-ESM2-0 | 2.45 | 0.93 | 0.74 | 83.95 | 89.61 | 83.95 | 89.36 |
| MRI-ESM2-0 | 5.85 | 0.92 | 0.67 | 76.54 | 90.62 | 90.13 | 76.54 |


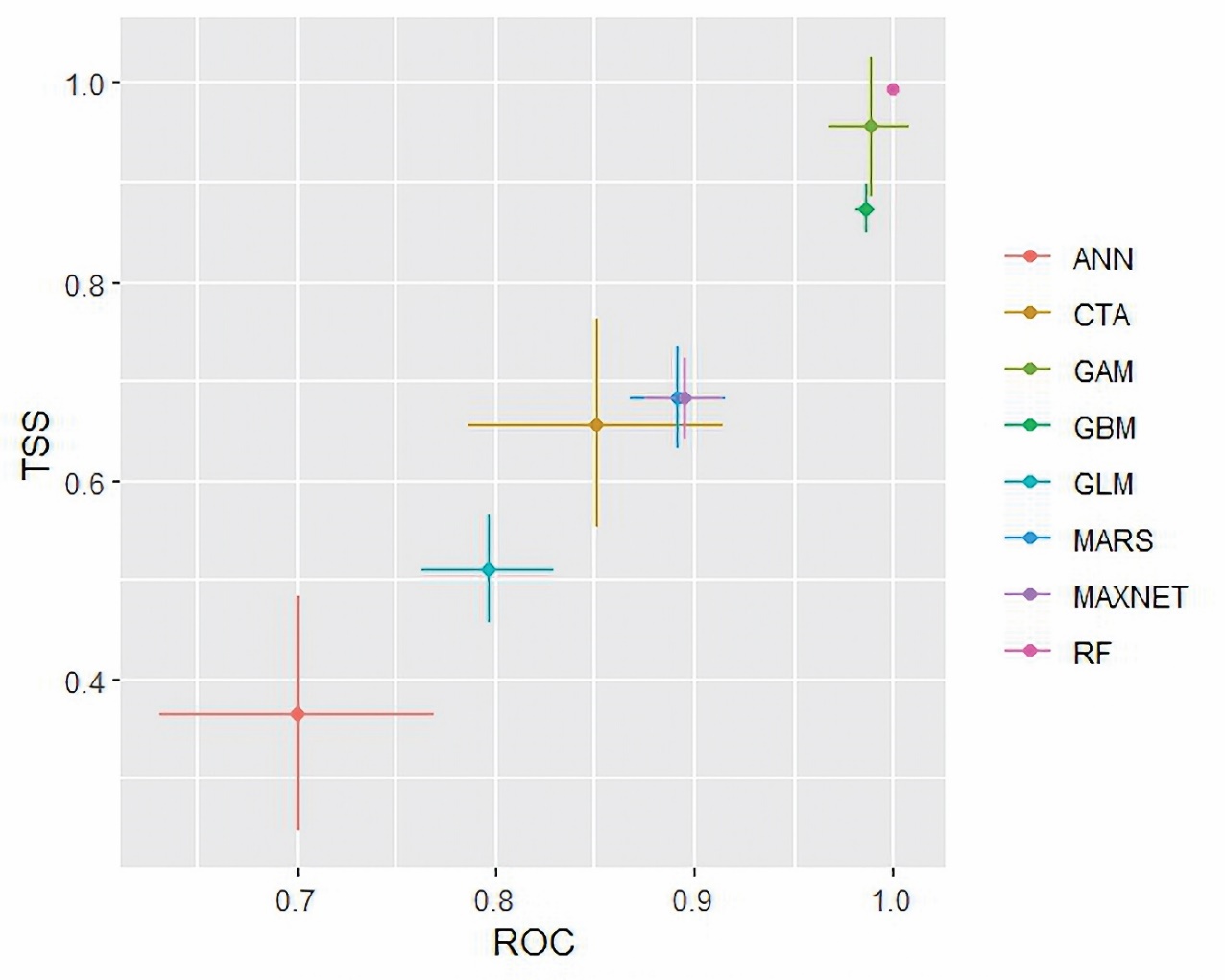


Fig. S1. Mean and range of predictive performance by receiver operating characteristic (ROC) curve and true skill statistic (TSS) over 800 model runs by eight individual modeling techniques (on the right). ANN = artificial neural networks, CTA = classification tree analysis, GAM = generalized additive model, GBM = generalized boosting model, GLM = generalized linear model, MARS = multivariate adaptive regression splines, MAXNET = maximum entropy, RF = random forest.


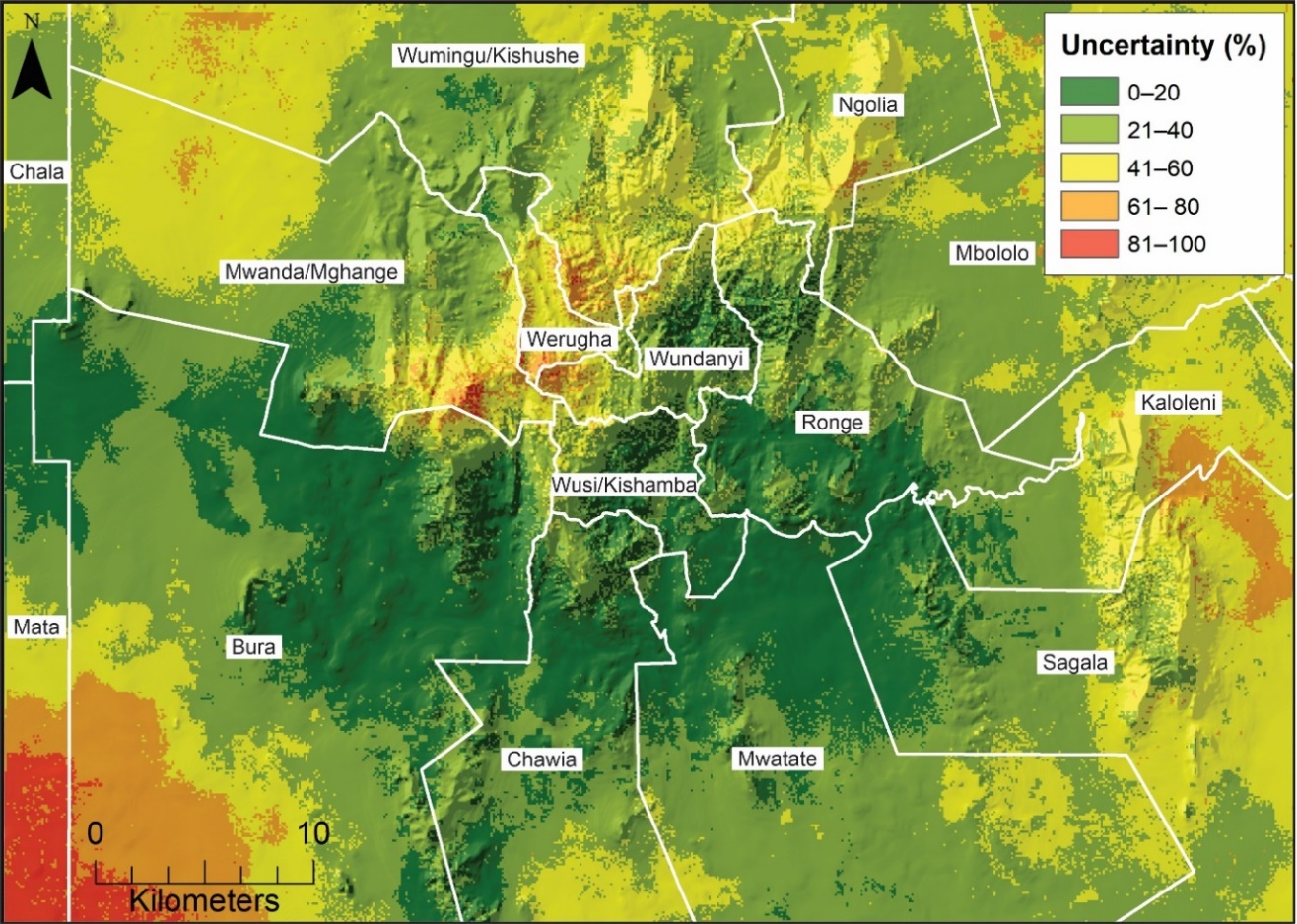


Fig. S2. Coefficient of variation in the prediction of current suitability for *Mops pumilus* estimating the uncertainty of the ensemble mean model over several modeling methods and presented by wards in the Taita Hills and surrounding plains. Uncertainty of the predictions was assessed by the coefficient of variation of predictions, where a high value indicates high uncertainty in predicted suitability (Thuiller et al., 2023).
